# Supplementary material for: Annotation-Based Gene-Peak Links Improve Regulatory Network Prediction of Gene Expression in Human Kidney Multi-Omics
Source: bioRxiv. 2026 Jun 17:2026.06.12.731741. Preprint. [Version 1] doi: 10.64898/2026.06.12.731741 (PMC13308000; doi:10.64898/2026.06.12.731741)
Supplement: Supplement 1 [file NIHPP2026.06.12.731741v1-supplement-1.pdf]

bioRxiv preprint doi: https://doi.org/10.64898/2026.06.12.731741; this version posted June 17, 2026. The copyright holder for this preprint (which was not certified by peer review) is the author/funder, who has granted bioRxiv a license to display the preprint in perpetuity. It is made available under a CC-BY 4.0 International license.

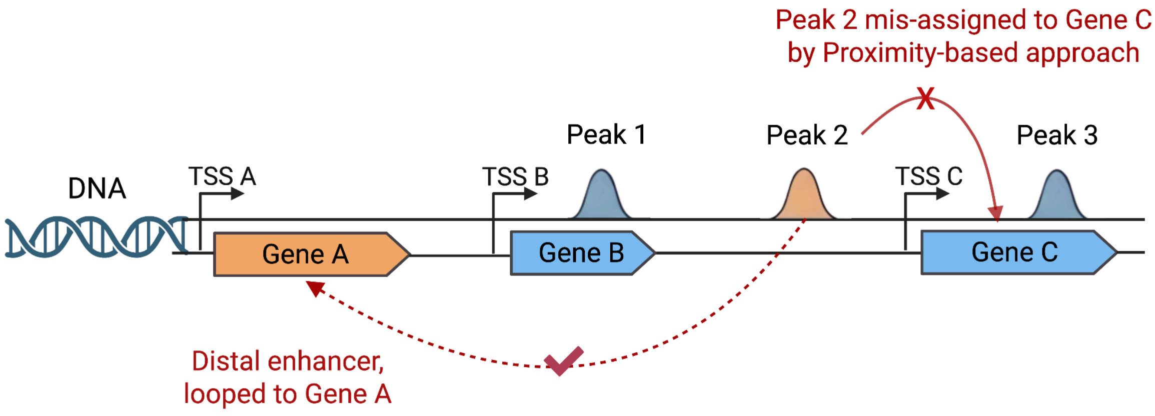

Supplementary Figure 1. Illustration of distal enhancer-gene interactions and potential misassignment by proximity-based peak-gene linkage

bioRxiv preprint doi: https://doi.org/10.64898/2026.06.12.731741; this version posted June 17, 2026. The copyright holder for this preprint (which was not certified by peer review) is the author/funder, who has granted bioRxiv a license to display the preprint in perpetuity. It is made available under a CC-BY 4.0 International license.

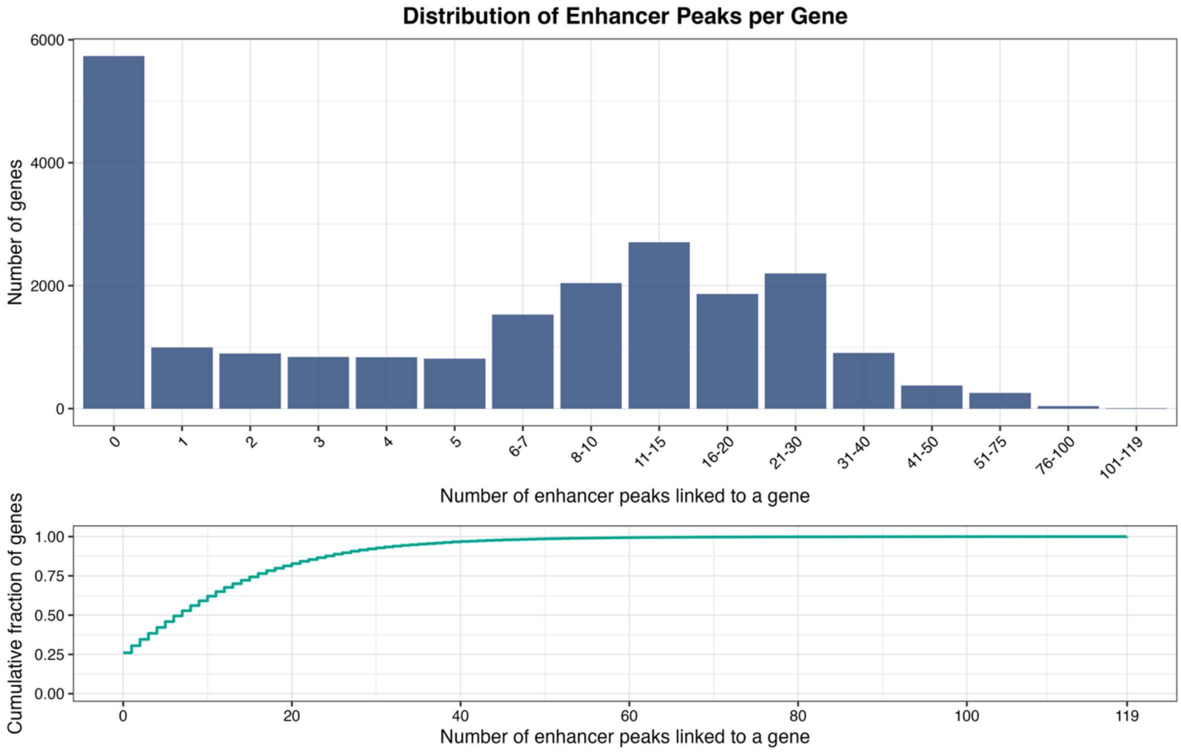

Supplementary Figure 2. Distribution of enhancer-based linked peaks per gene across all 22,035 genes

bioRxiv preprint doi: https://doi.org/10.64898/2026.06.12.731741; this version posted June 17, 2026. The copyright holder for this preprint (which was not certified by peer review) is the author/funder, who has granted bioRxiv a license to display the preprint in perpetuity. It is made available under a CC-BY 4.0 International license.

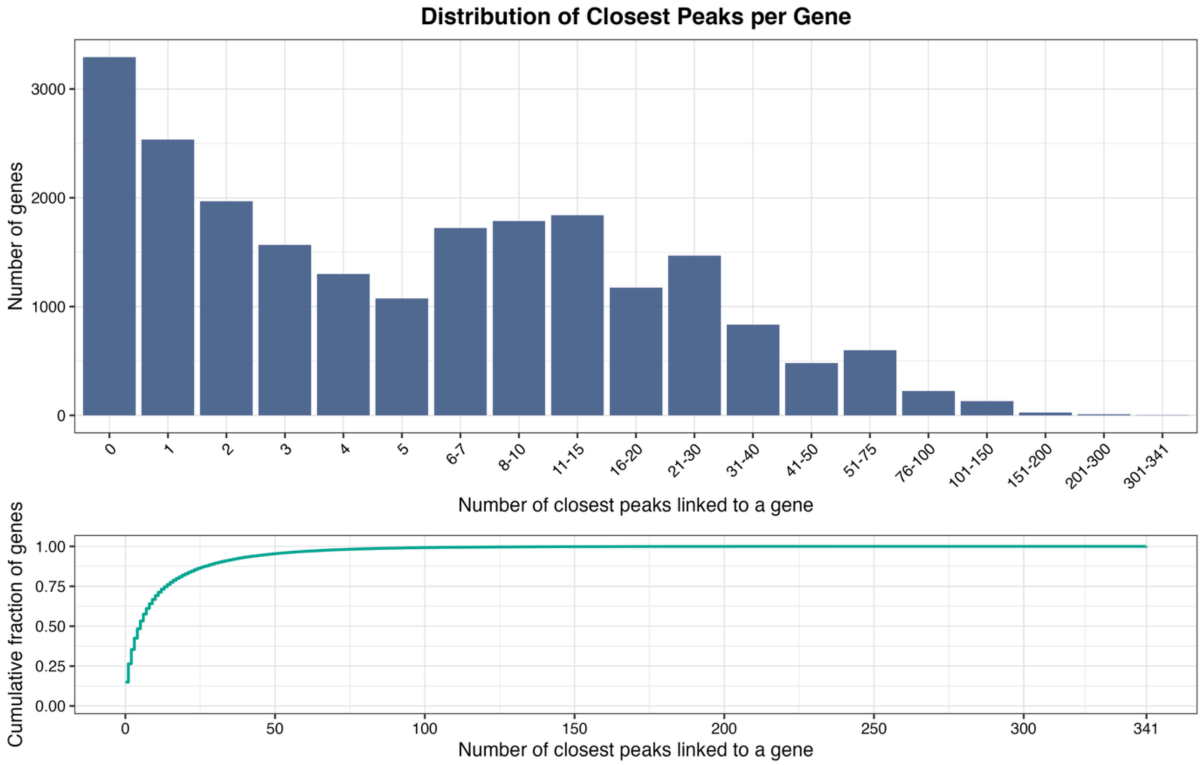

Supplementary Figure 3. Distribution of proximity-based linked peaks per gene across all 22,035 genes

bioRxiv preprint doi: https://doi.org/10.64898/2026.06.12.731741; this version posted June 17, 2026. The copyright holder for this preprint (which was not certified by peer review) is the author/funder, who has granted bioRxiv a license to display the preprint in perpetuity. It is made available under a CC-BY 4.0 International license.

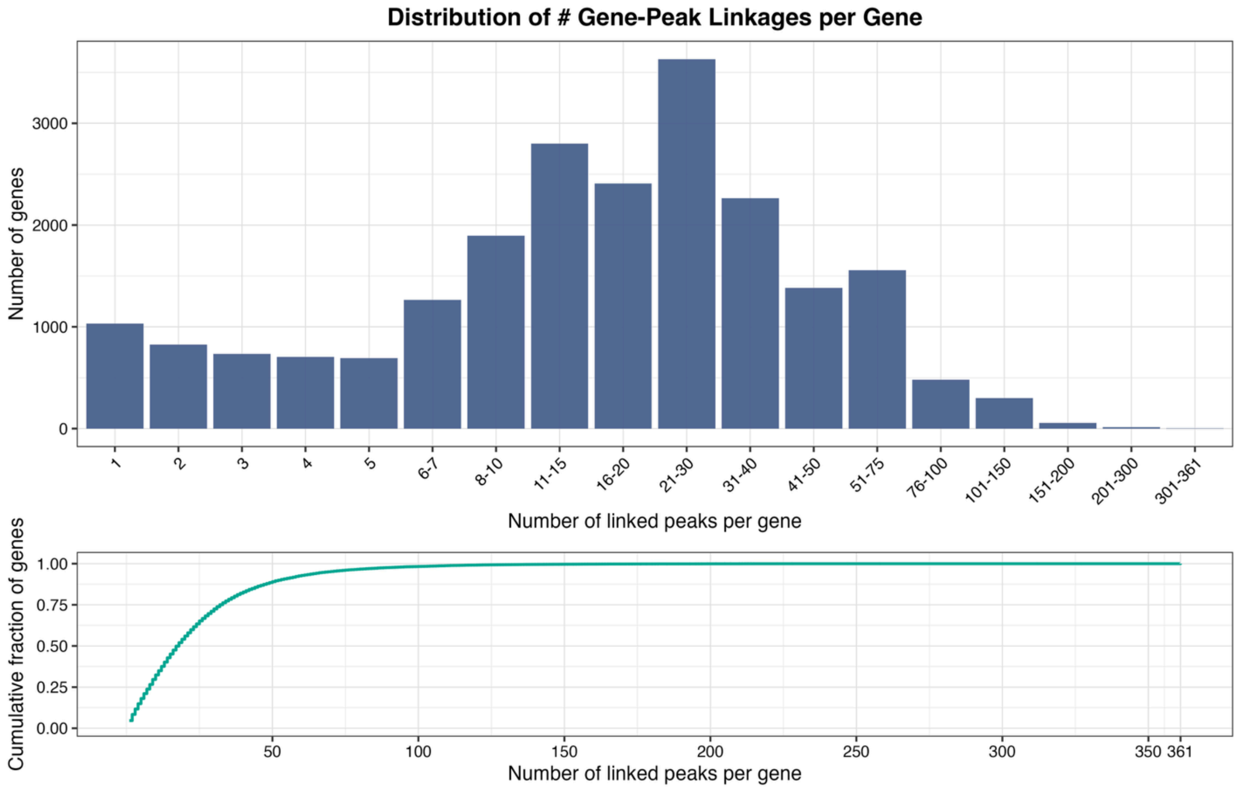

Supplementary Figure 4. Distribution of gene-peak linkage counts per gene.

bioRxiv preprint doi: https://doi.org/10.64898/2026.06.12.731741; this version posted June 17, 2026. The copyright holder for this preprint (which was not certified by peer review) is the author/funder, who has granted bioRxiv a license to display the preprint in perpetuity. It is made available under a CC-BY 4.0 International license.

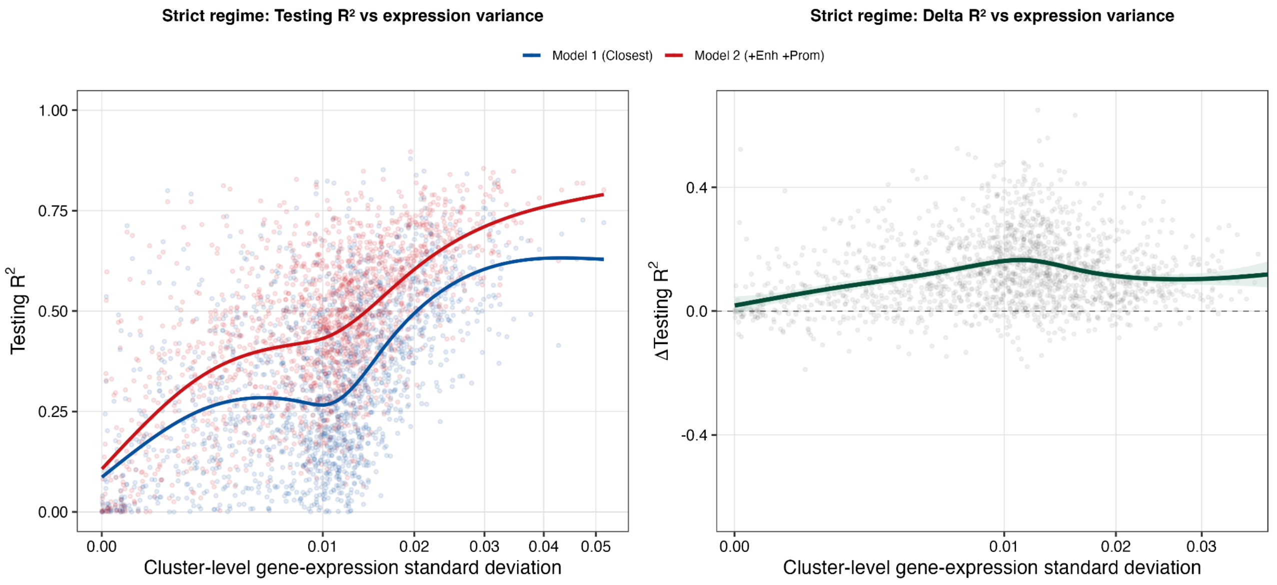

Supplementary Figure 5. Association of model prediction performance with cluster-level gene-expression variance in the strict regime. Left panel: testing R^2 for Model 1 and Model 2 as a function of cluster-level gene-expression variance. Right panel: per-gene improvement in predictive performance ($\Delta R^2 = R^2$ of Model 2 - R^2 of Model 1) as a function of cluster-level gene-expression variance.

bioRxiv preprint doi: https://doi.org/10.64898/2026.06.12.731741; this version posted June 17, 2026. The copyright holder for this preprint (which was not certified by peer review) is the author/funder, who has granted bioRxiv a license to display the preprint in perpetuity. It is made available under a CC-BY 4.0 International license.

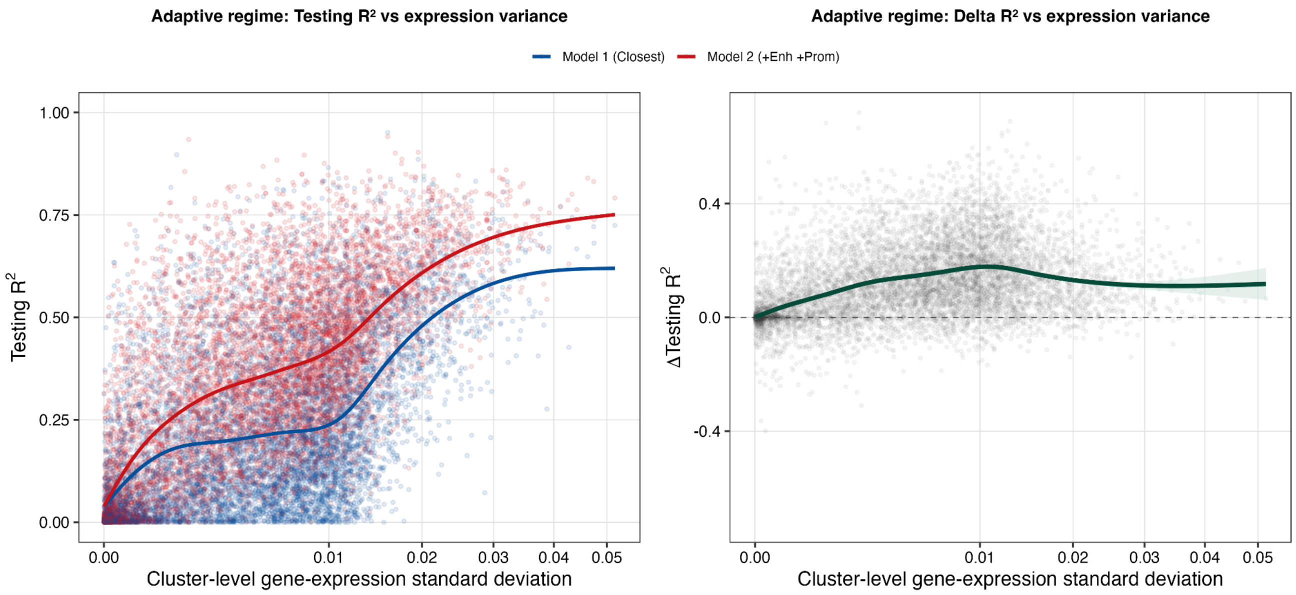

Supplementary Figure 6. Association of model prediction performance with cluster-level gene-expression variance in the adaptive regime. Left panel: testing R^2 for Model 1 and Model 2 as a function of cluster-level gene-expression variance. Right panel: per-gene improvement in predictive performance ($\Delta R^2 = R^2$ of Model 2 - R^2 of Model 1) as a function of cluster-level gene-expression variance.
